# Electrochemical characteristics of amorphous silicon carbide film as a lithium-ion battery anode†

**DOI:** 10.1039/c7ra12463e

**Published:** 2018-01-30

**Authors:** X. D. Huang, F. Zhang, X. F. Gan, Q. A. Huang, J. Z. Yang, P. T. Lai, W. M. Tang

**Affiliations:** Key Laboratory of MEMS of the Ministry of Education, Southeast University Nanjing 210096 China xdhuang@seu.edu.cn; School of Chemistry and Chemical Engineering, Nanjing University of Science and Technology Nanjing 210094 China jiazhiyang@sina.com; Department of Electrical and Electronic Engineering, The University of Hong Kong Hong Kong China; Department of Applied Physics, The Hong Kong Polytechnic University Hong Kong China wm.tang@polyu.edu.hk

## Abstract

The electrochemical reactions of SiC film with Li^+^ have been investigated by electrochemical characterization and X-ray photoelectron spectroscopy. The SiC film is prepared by inductively-coupled-plasma chemical-vapor-deposition (ICP-CVD) technique and displays an amorphous state due to the low processing temperature (∼350 °C). An irreversible reaction of SiC with Li^+^ occurs with the formation of lithium silicon carbide (Li_*x*_Si_*y*_C) and elemental Si, followed by a reversible alloying/dealloying reaction of the elemental Si with Li^+^. The 500 nm SiC film shows an initial reversible specific capacity of 917 mA h g^−1^ with a capacity retention of 41.0% after 100 cycles at 0.3C charge/discharge current, and displays much better capacity retention than the Si film (5.2%). It is found that decreasing the SiC thickness effectively improves the specific capacity by enhancing the reaction kinetics but also degrades the capacity retention (for 250 nm SiC, its initial capacity is 1427 mA h g^−1^ with a capacity retention of 25.7% after 100 cycles). The better capacity retention of the 500 nm SiC anode is mainly because residual SiC exists in the film due to its incomplete reaction caused by its lower reaction kinetics, and it has high hardness and can act as a buffer matrix to alleviate the anode volume change, thus improving the mechanical stability and capacity retention of the SiC anode.

## Abstract 2

The electrochemical reactions of SiC film with Li^+^ have been investigated by electrochemical characterization and X-ray photoelectron spectroscopy.

## Graphical abstract

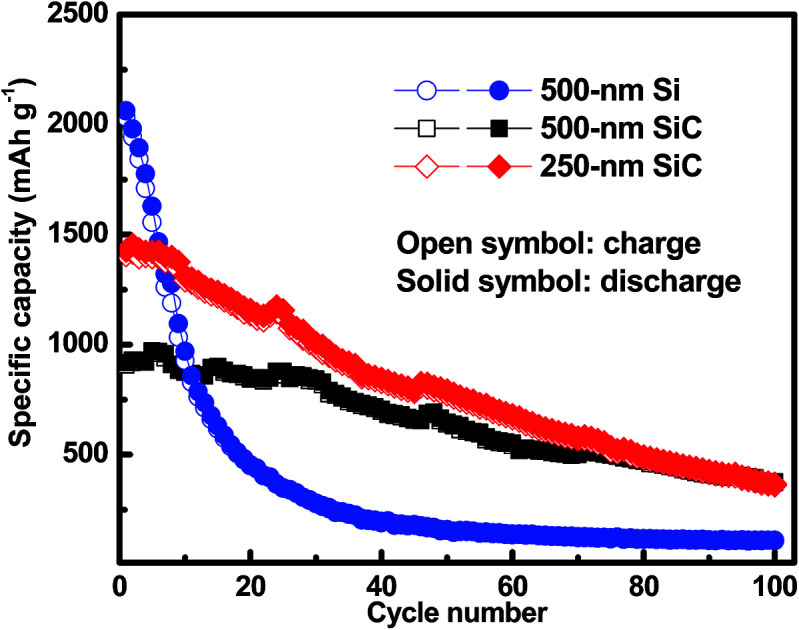

## 1. Introduction

The developments of microelectronics and MEMS (micro-electro-mechanical systems) demand micro-sized on-board power sources for establishing an autonomous microsystem.^1–5^ A thin-film lithium-ion battery (LIB) is promising for on-board power supply because of its high energy and power densities. Compared with the conventional carbon anode (*ca.* 372 mA h g^−1^), Si seems to be more suitable as the LIB anode due to its ultrahigh theoretical capacity (*ca.* 4200 mA h g^−1^).^4–13^ Moreover, Si-based materials (*e.g.* Si, silicon oxide, silicon nitride and silicon carbide) are widely used in microelectronics; consequently, LIBs with Si-based materials as the anode are easy to integrate with electronic and MEMS devices.^4,5^ This is helpful to minimize the size of the autonomous microsystem as well as reduce the fabrication cost. The main issue of the Si anode is its poor capacity retention due to severe volume change during electrochemical charge (delithiation) and discharge (lithiation) cycling.^6–13^ As alternatives, silicon oxide and silicon nitride films can achieve considerable capacity as well as good capacity retention,^14–23^ thus having receiving much attention. Moreover, it has been demonstrated that C introduced to Si (in the form of Si–C composite or as an encapsulation layer on the Si surface) is effective to accommodate the volume change and thus improve the LIB capacity retention.^24–28^ However, the formation of silicon carbide (SiC) easily happens when C is in contact with Si due to the strong bonding tendency between them.^24,25^ Unlike silicon oxide and silicon nitride,^16–23^ since SiC has been regarded as an inactive anode material for LIB, many efforts have been made to prohibit the formation of SiC during introducing C to Si, which inevitably complicate the fabrication process and cost.^24–30^ Until recently, both theoretical and experimental results have demonstrated that SiC can act as a LIB anode and achieve a high reversible capacity (*e.g.*, 1200 mA h g^−1^ over 200 cycles at a C/30 current rate in ref. 33).^31–35^ Consequently, SiC has received an increasing interest in recent years. However, little work has been paid to the electrochemical reaction mechanisms of the SiC anode with Li^+^.^32,33^ Zhang *et al.* reported that the reaction mechanisms of the SiC film with Li^+^ follow the conversion reaction; that is, conversion reaction from SiC to elemental Si occurs reversibly, followed by a reversible alloying/dealloying reaction of Si with Li^+^.^32^ According to the above reaction mechanisms, the capacity of the SiC anode is calculated to be 5626 mA h g^−1^, which is much higher than its experimental value (309 mA h g^−1^).^32^ Therefore, the mechanisms are still unclear and need to be carefully investigated. So far, SiC used in the LIB is usually in the form of nanostructure (*e.g.*, nanoparticle and nanowire);^33–35^ for comparison, the primary form of SiC in the microelectronics is film. Compared with the nanostructured SiC, advantages of the SiC film include better uniformity, higher deposition rate, more compatibility with the microelectronic process and thus easier integration with electronic and MEMS devices. It is known that the form of the LIB anode has a great influence on its electrochemical characteristics, for instance, the nanostructured anode usually has higher surface area and higher activity than the film counterpart. Therefore, even though the nanostructured SiC has been demonstrated to be a promising anode material, the feasibility of SiC film as the anode still needs to be carefully investigated.

## 2. Experimental

### 2.1 Preparation of the anode films

500 nm SiC film was deposited on stainless-steel substrate by inductively-coupled-plasma chemical-vapor-deposition (ICP-CVD, Oxford 380) technique at 350 °C, which is the common technique for SiC deposition in the microelectronics.^36^ During the deposition, Ar, SiH_4_ and CH_4_ were used as precursors and their flow rates were 30 sccm, 30 sccm and 45 sccm respectively, and the deposition rate of the film is about 18 nm min^−1^. For practical applications, a relatively thick SiC film is desirable for improving the anode capacity; however, in order to investigate the effects of the film thickness on the electrochemical performance of the SiC anode, a thin (*ca.* 250 nm) SiC film was also prepared. For comparison, 500 nm Si film was also prepared on stainless-steel substrate by sputtering using a pure Si target at room temperature, followed by a thermal annealing at 350 °C in an Ar ambient. The thermal annealing was adopted to ensure that the Si film possessed similar thermal budget as the SiC one.

### 2.2 Characterization

The thickness of the films was measured by using ellipsometry (Horiba Uvisel). For electrochemical characterization, the SiC and Si electrodes were assembled into CR2016-type coin cells with Celgard 2400 as separator, 1 M LiPF_6_ dissolved in ethylene carbonate/dimethyl carbonate (v/v = 1 : 1) as electrolyte, and Li foil as counter electrode. Cyclic voltammetry was recorded at a scanning rate of 0.1 mV s^−1^ between 0 V and 3 V *versus* Li/Li^+^ (CHI 660E). Galvano-static cycling was carried out between 0.01 V and 3.0 V *versus* Li^+^/Li (Land CT2001A). To examine the film properties after electrochemical cycling, the anode electrodes were took out from the coin cells after 100 charge/discharge cycles and dried in an Ar ambient. The crystalline structures of the films were investigated by X-ray diffraction (XRD, Bruker D8 Advance). The morphologies of the films were characterized by scanning electron microscopy (SEM, Zeiss Ultra Plus). The elemental compositions of the films were evaluated by energy dispersive X-ray spectrometry (EDX). The chemical states of the films were measured by X-ray photoelectron spectroscopy (XPS, PHI Quantera II) and the XPS penetration depth is about 10 nm. The XPS spectra were calibrated according to ref. 37. During the XPS measurements, the depth profiling was performed by Ar^+^ sputtering with an etching rate of about 3.5 nm min^−1^.

## 3. Results and discussion

**Fig. 1:**
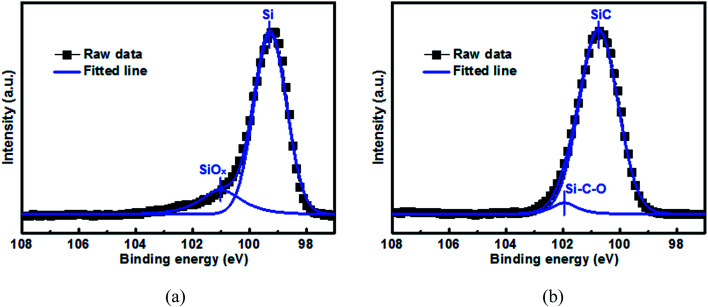
Si 2p XPS spectrum for the 500 nm (a) Si anode film and (b) SiC anode film before electrochemical cycling. The samples are sputtered by Ar^+^ for 1 min before test.

Fig. 1 shows the Si 2p XPS spectrum as well as the curve-fitting lines for the 500 nm Si and SiC samples before electrochemical cycling. For the Si sample, its spectrum consists of one intense peak at 99.3 eV and one weak peak at 101.0 eV, which can be assigned to elemental Si and SiO_*x*_ respectively.^37^ SiO_*x*_ is formed due to the oxidation reaction of elemental Si with oxygen during the film preparation. For comparison, the spectrum for the SiC sample mainly consists of one intense peak at 100.7 eV and one weak peak at 102.0 eV, which agree well with SiC and Si–C–O respectively.^38^ The formation of Si–C–O can be further confirmed by the C 1s spectrum as shown in Fig. S1.† Note that no elemental Si component is observed in the SiC sample. The elemental compositions of the Si and SiC films can be determined by EDX (as seen in Fig. S2†). It is found that both the Si and SiC films consist of Si, C and O elements. For the Si film, the relative atomic content of the Si, O and C elements is 72.9%, 5.6% and 21.5% respectively. The C element in the Si film is mainly due to the surface contamination and is mainly located at the film surface, and these can be demonstrated by the C 1s XPS depth profiling as shown in Fig. S3.† For the SiC film, the atomic content of the Si, C and O elements is 49.8%, 45.7% and 4.5% respectively, where the atomic ratio between the Si and C elements is close to the ideal stoichiometric ratio. It is worth mentioning that the SiC film has a good thermal stability, which results in its much lower O content (∼5.6%) than the Si film (∼21.5%) even though both films go through similar thermal budget. It has been reported that both SiO_*x*_ and Si–C–O can act as an active anode and have much better capacity retention than the Si anode.^15–18,38^ However, in this work, although the SiO_*x*_ content (∼15.6% from Fig. 1(a)) in the Si film is much higher than the Si–C–O content (4.1% from Fig. 1(b)) in the SiC film, the SiC sample displays much better capacity retention than the Si sample (as seen in Fig. 6 and the details will be discussed later), suggesting that the SiC component plays the dominant role in the anode capacity retention as well as the anode reaction.

**Fig. 2:**
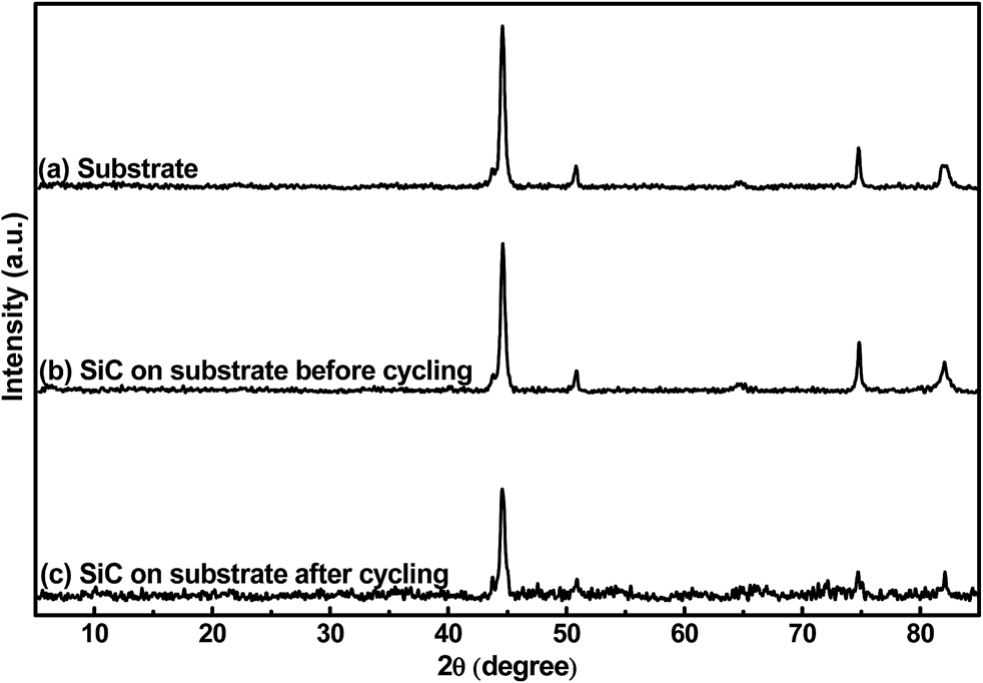
XRD patterns of (a) the stainless-steel substrate, (b) 500 nm SiC on the substrate before cycling and (c) 500 nm SiC on the substrate after cycling.

Fig. 2 shows the crystalline patterns of the stainless-steel substrate and the 500 nm SiC film on the substrate before and after electrochemical cycling. It is found that the samples display same XRD patterns, suggesting that all the patterns are from the substrate and the SiC film (both before and after cycling) is amorphous. The amorphous state of the SiC film should be mainly ascribed to its low thermal processing temperature. Due to its much higher plasma density of the ICP-CVD technique, ICP-CVD can adopt much lower thermal processing temperature for the SiC deposition than other techniques (*e.g.* microwave-plasma CVD and low-pressure CVD, ≥1000 °C).^26^ It is worth pointing out that this lower processing temperature is quite beneficial to suppress the damages to electronic and MEMS devices when they are fabricated together with the LIB, thus helpful to develop an autonomous microsystem. In addition, regarding the materials used as the LIB electrode, it has been reported that the reactions of the amorphous material with Li^+^ has better reversibility than the reactions of the crystalline counterpart with Li^+^.^39^ Also, the amorphous material would form percolation pathways to facilitate Li^+^ diffusion; consequently, the amorphous material displays higher reaction kinetics than the crystalline counterpart.^40,41^ Furthermore, due to its homogeneous nature, the amorphous material has higher ability to endure the stress induced by the electrode volume changes during repeated electrochemical cycles than the crystalline one; therefore, the former exhibits better capacity retention than the latter.^41,42^ Based on the above hints, the amorphous SiC anode film is expected to obtain better electrochemical performance than the crystalline counterpart.

**Fig. 3:**
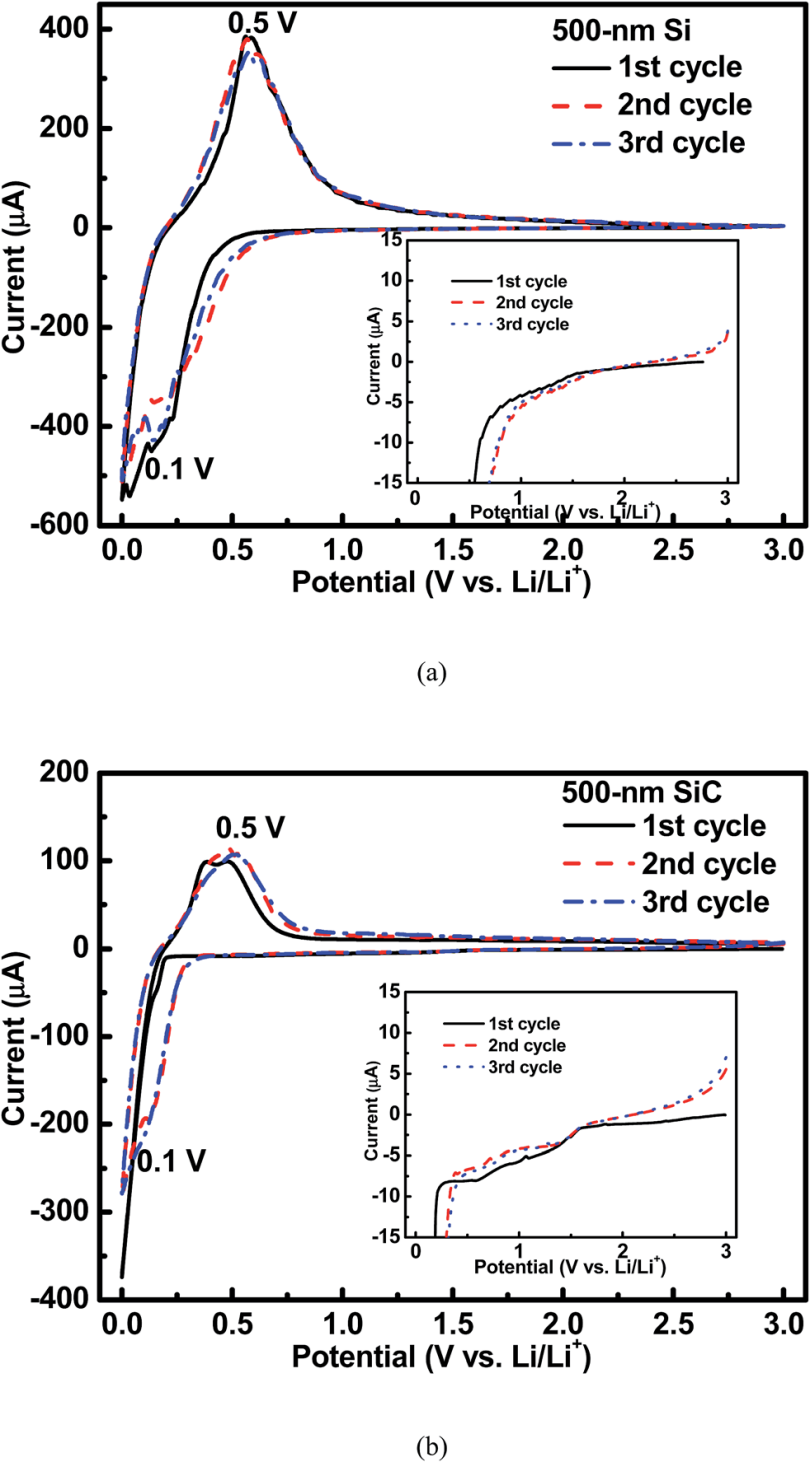
CV profiles of the 500 nm (a) Si sample and (b) SiC sample. The inset shows that there is an extra cathodic peak between 0.5–1.5 V for the SiC sample compared with the Si one.

Fig. 3 shows the cyclic voltammetry (CV) curves of the 500 nm Si and SiC samples. As seen in Fig. 3(a), for the Si sample, an obvious cathodic peak at around 0.1 V and an obvious anodic peak at around 0.5 V can be clearly observed, which agree well with the reversible alloying/dealloying reaction of Si with Li^+^.^7–10^ As shown in Fig. 3(b), this pair of redox peaks can be also found in the SiC sample. However, for the SiC sample, the cathodic peak at around 0.1 V is very weak in the 1^st^ CV cycle mainly due to its negligible Si content (as seen in Fig. 1(b)) and then becomes much stronger in the following cycles, suggesting that more Si are formed by SiC to participate in the alloying/dealloying reaction. In addition, as seen in the inset of Fig. 3, for the SiC sample, its CV curve in the 1^st^ cycle displays a broad cathodic peak between 0.5 V and 1.5 V compared with that for the Si sample, indicating the existence of an extra electrochemical reaction in the SiC sample. This peak becomes unclear with cycling, suggesting that the reaction associated with this peak is irreversible and mainly happens in the initial cycle.

**Fig. 4:**
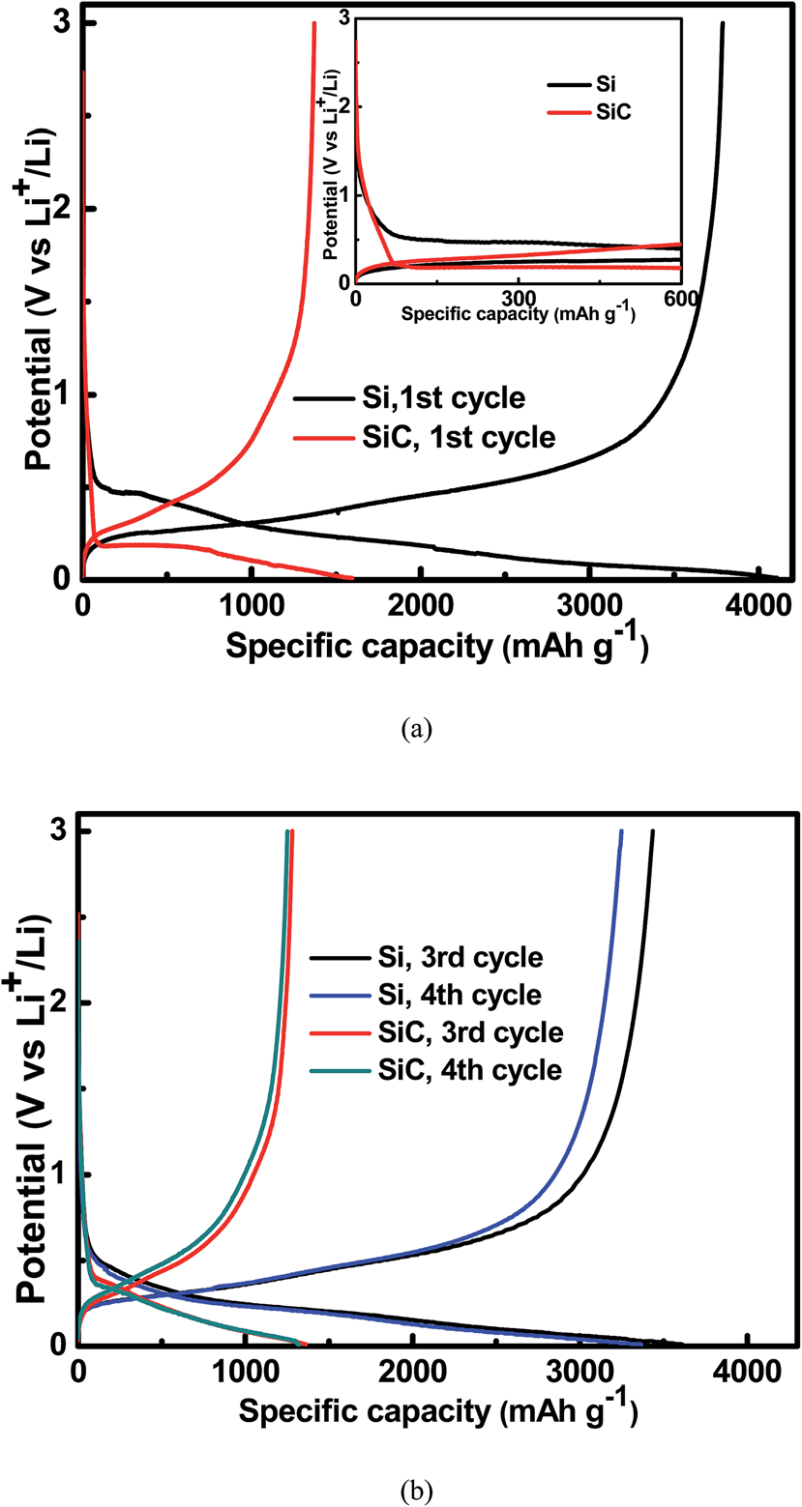
Charge/discharge voltage profiles for the 500 nm Si and SiC and samples at a current density of 0.01C (a) in the 1^st^ cycle; (b) in the 3^rd^ and 4^th^ cycles. The inset of Fig. 3(a) shows that the SiC sample displays a slope in the range of 0.5–1.5 V followed by a voltage overshoot at the initial stage of the 1^st^ discharge process.

Fig. 4 shows the charge/discharge voltage profiles of the 500 nm Si and SiC samples at a current density of 0.01C. As seen in the inset of Fig. 4(a), compared with the Si sample, the SiC sample displays a slope in the range of 0.5–1.5 V followed by a voltage overshoot at the initial stage of the first discharge process. This is a typical characteristic of a reaction with nucleation of a new phase, indicating that significant change occurs in the SiC anode during the first discharge process.^21–23^ Both the slope and overshoot disappear and the SiC sample displays similar voltage profiles to the Si sample in the following cycles (as shown in Fig. 4(b)), suggesting similar electrochemical reactions between them in the following cycles. The Si and SiC samples display an initial charge/discharge capacity of 3787 mA h g^−1^/4110 mA h g^−1^ and 1371 mA h g^−1^/1595 mA h g^−1^, corresponding to a coulombic efficiency (CE) of 92.1% and 86.0% respectively. The SiC sample displays lower initial capacity and CE than the Si one. This should be due to the extra irreversible reaction happening in the SiC anode as discussed above and the details will be discussed later. It is worth pointing out that both the CV and charge/discharge voltage profiles of the SiC anode film in this work are quite different from those of the nanostructured SiC in the literatures, which have reported that the reactions of SiC with Li^+^ do not involve the formation of Li–Si alloy.^33–35^ This suggests different electrochemical reactions between them.

In the ref. 32, the reaction mechanisms of the SiC film with Li^+^ is expressed by

1 SiC + 4Li^+^ + 4e^−^ ⇔ Li_4_C + Si

2 Si + 4.4Li^+^ + 4.4e^−^ ⇔ Si_4.4_Li

According to the above equations, conversion reaction from SiC to elemental Si occurs reversibly, followed by a reversible alloying/dealloying reaction of Si with Li^+^. The corresponding SiC capacity can be calculated to be 5626 mA h g^−1^, which is much higher than the test value (309 mA h g^−1^).^32^ It has been reported that the conversion reaction shown in the eqn (1) can be reversible or irreversible.^21,32,42^ As discussed earlier, there is an irreversible reaction in the initial cycle for the SiC sample, which leads to the formation of elemental Si (and thus higher capacity of the sample) in the following cycles. Therefore, given that the reaction shown in the eqn (1) is irreversible, the corresponding coulombic efficiency of the SiC anode in the initial cycle is calculated to be 47.6%, which is much lower than the actual value (86.0%) in this work. Therefore, according to the above analysis, the conversion reaction is not appropriate to describe the reaction mechanisms of SiC with Li^+^ in this work; alternatively, we suggest that the reactions of SiC with Li^+^ should be followed by

3 SiC + *x*Li^+^ + *x*e^−^ ⇒ Li_*x*_Si_*y*_C + (1 − *y*)Si (y < 1)

4 Si + 4.4Li^+^ + 4.4e^−^ ⇔ Si_4.4_Li

**Fig. 5:**
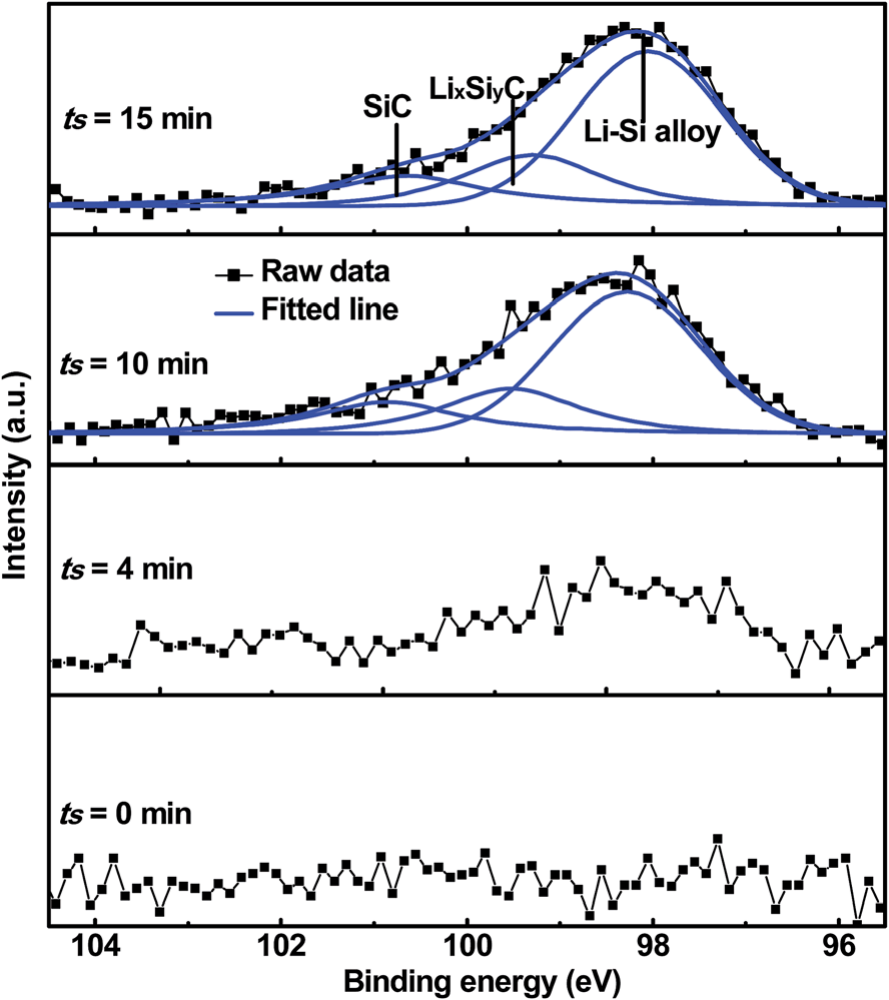
Si 2p XPS spectrum as a function of Ar^+^ sputtering time for the 500 nm SiC anode film at a fully discharge (0.01 V) state.

Due to the strong covalent bonding between Si and C, Li_*x*_Si_*y*_C has higher formation energy than Li_4_C, which leads to that change in the Gibbs free energy (Δ*G*) caused by the eqn (3) should be more negative than Δ*G* caused by the eqn (1). Therefore, the eqn (3) is more likely to occur spontaneously than the eqn (1). To gain more insight into the electrochemical reactions, the Si 2p XPS spectrum as a function of Ar^+^ sputtering time (*t*_s_) is investigated for the SiC anode at a fully discharge state. As shown in Fig. 5, when *t*_s_ = 0 s, no Si spectrum is observed mainly due to the formation of a SEI (solid electrolyte interphase) layer on the sample surface. When *t*_s_ = 4 min, a weak Si spectrum begins to appear. The appearance of the Si spectrum at *t*_s_ = 4 min suggests that the SEI thickness is about 10 nm. The SEI layer in this work is thinner than that (∼40 nm) for the nanostructured anode in the literatures,^37^ thus contributing to the higher coulombic efficiency of the anode in this work.^32,33,37^ The spectrum intensity increases with increasing *t*_s_ and then tends to be stable as *t*_s_ ≥ 10 min. Compared with the Si 2p spectrum before cycling (as seen in Fig. 1(b), the full width at half maximum (FWHM) is 1.7 eV), the spectrum (as *t*_s_ ≥ 10 min) after cycling are significantly broadened (FWHM ∼ 2.8 eV), suggesting more complex components formed in the anode after cycling. In addition, compared with the spectrum (∼100.7 eV) before cycling, the spectrum at the discharge state makes a negative shift to 98.1 eV, which can be assigned to Li–Si alloy formed by the alloying reaction of Si with Li^+^.^37^ Moreover, for the Si 2p spectrum at the discharge state, besides the dominant component at 98.1 eV, two extra weak components at 99.5 eV and 100.7 eV are also observed, corresponding to Li_*x*_Si_*y*_C and SiC respectively. Li_*x*_Si_*y*_C is formed by the irreversible reaction of SiC with Li^+^ as shown in the eqn (3). This irreversible reaction with the formation of Li_*x*_Si_*y*_C would consume Li^+^, thus resulting in the lower coulombic efficiency of the SiC anode than the Si one in the initial cycle as mentioned in Fig. 4. The existence of SiC at the fully discharge state suggests that there is some residual SiC in the anode not participating in the reactions, thus contributing to the lower capacity of the SiC sample than the Si one. According to Fig. 5, the relative atomic content of the Si–Li alloy, Li_*x*_Si_*y*_C and SiC components in the anode (as *t*_s_ ≥ 10 min) is about 61.2%, 20.5% and 18.3% respectively. It is noted that among each component in the anode, only the formation of Si–Li alloy contributes to the anode reversible capacity. Combining the SiC charge capacity in the initial cycle (∼1400 mA h g^−1^ from Fig. 4) with the relative content of the Si–Li alloy component in the anode (∼61.2% from Fig. 5), the value of *y* in the eqn (3) can be calculated to be 0.22. Moreover, regarding the coulombic efficiency of the SiC anode in the initial cycle (∼86.0% from Fig. 4), the value of *x* and *y* in the eqn (3) should meet the following expression5
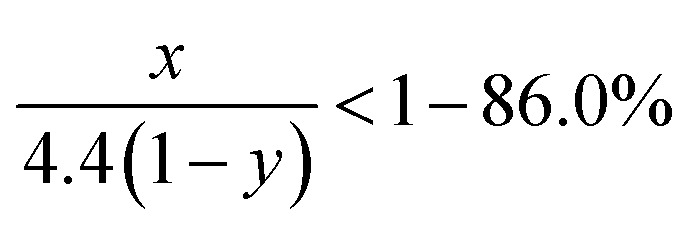


According to the above expression, the value of *x* should be less than 0.48. Moreover, as discussed earlier, the irreversible reaction (3) induces a slope in the range of 0.5–1.5 V at the initial stage of the first discharge process (as seen in Fig. 4(a)) and the corresponding irreversible capacity of this slope is about 60 mA h g^−1^ according to Fig. 4(a). Combining this irreversible capacity with the relative Li_*x*_Si_*y*_C content (∼20.5% from Fig. 5) in the anode, the value of *x* can be determined to be 0.44. This extracted *x* value satisfies the expression (5) well. It is noted that this irreversible capacity only accounts for a tiny portion of the whole capacity (∼1595 mA h g^−1^ as seen in Fig. 4(a)), leading to that the features corresponding to this irreversible reaction is not obvious in the CV and charge/discharge curves.

**Fig. 6:**
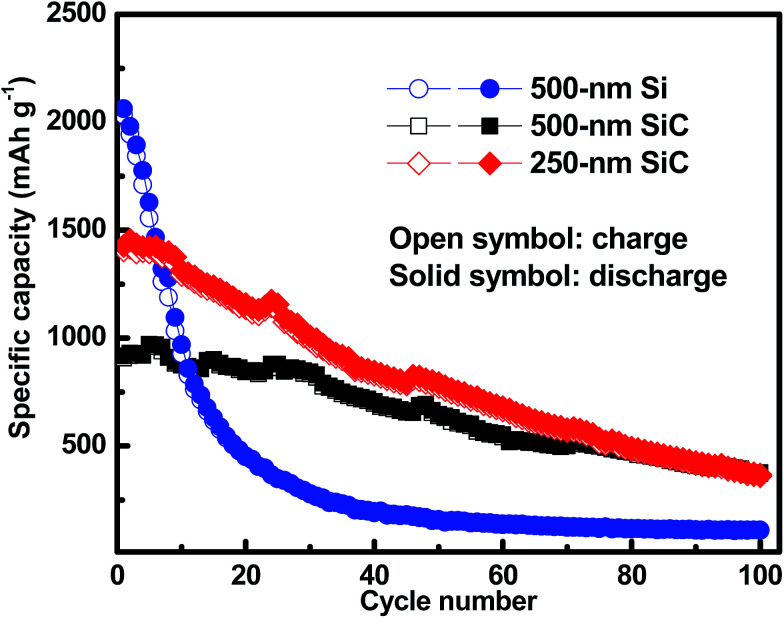
Galvano-static cycling of the Si, thin SiC and thick SiC samples at a current density of 0.3C. The samples are activated at a low current density of 0.01C before test.

**Fig. 7:**
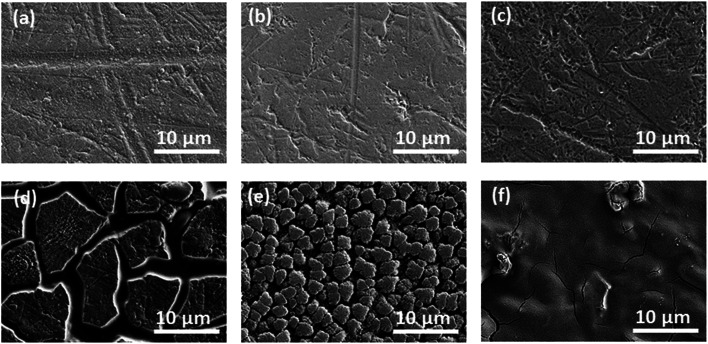
SEM images of the samples before cycling: (a) 500 nm Si, (b) 250 nm SiC and (c) 500 nm SiC; SEM images of the samples after cycling: (d) 500 nm Si, (e) 250 nm SiC and (f) 500 nm SiC.

Fig. 6 shows the cycling characteristics of the Si and SiC samples at a 0.3C charge/discharge current, and both display a high CE (>98.0%) after going through several cycles. For the 500 nm SiC sample, the initial discharge reversible capacity is 917 mA h g^−1^ and degrades gradually to 376 mA h g^−1^ after 100 cycles, corresponding to a capacity retention of 41.0%. For comparison, the initial capacity of the Si sample is 2062 mA h g^−1^, and its capacity decreases drastically at first and then remains constant with cycling, corresponding to a capacity retention of 5.2% after 100 cycles. It is obvious that the SiC sample exhibits much better capacity retention than the Si one. The SiC sample displays lower specific capacity in the initial cycles than the Si one partially due to its incomplete reaction as demonstrated in Fig. 5. For practical applications, relatively thick SiC and Si films (∼500 nm) are utilized for improving the anode capacity in this work. However, the thick anode film would reduce the anode conductivity (and thus the reaction kinetics). This has more severe influence on the SiC anode than the Si one mainly because of its lower activity than the latter,^25,29,30^ thus resulting in residual SiC component in the SiC sample even after repeated cycling (as seen in Fig. 5). As demonstrated in Fig. 6, decreasing the SiC thickness is effective to enhance the reaction kinetics and thus increase the specific capacity (the initial capacity is 1427 mA h g^−1^ and 917 mA h g^−1^ for the 250 nm and 500 nm SiC respectively).^21,23^ The capacity retention for the thin SiC anode is 25.7% after 100 cycles. One interesting phenomenon is that the thin SiC anode (∼25.7%) displays worse capacity retention than the thick one (∼41.0%). It is known that a thinner anode film has stronger ability to suppress the stress induced material degradation caused by repeated cycling; consequently, the thin anode film usually exhibits better capacity retention than the thick one.^6,21,23^ However, in this work, the better capacity retention for the thick SiC sample than the thin one suggests that the residual SiC component (caused by the incomplete reaction) in the anode film plays a critical role in improving the capacity retention. The SiC has very high hardness (*ca.* 18 GPa *vs.* 11 GPa for Si),^43,44^ which can act as a buffer matrix surrounding the active anode materials, thus suppressing the volume change as well as its induced stress during repeated cycling. It is noted that the film hardness would decrease rapidly after Li^+^ insertion into the film;^45,46^ therefore, it is believed that the Li_*x*_Si_*y*_C component formed by the irreversible reaction shown in (3) has less contribution to the capacity retention than the SiC component due to its lower hardness. Fig. 7 exhibits the SEM images of the Si, thin SiC and thick SiC samples before and after cycling. The Si and thin SiC anodes after cycling break into pieces mainly because of its severe volume change induced by the repeated cycling, thus leading to their low capacity retention. On the contrary, the thick SiC film after cycling is almost intact with only a few hairline cracks, demonstrating that the thick SiC film has much better mechanical stability, thus resulting in its better capacity retention.

## 4. Conclusion

500 nm SiC film prepared by ICP-CVD is investigated as the LIB anode by comparison with the Si film. The SiC film is amorphous due to the low processing temperature, which is beneficial for the anode performance. An irreversible reaction of SiC with Li^+^ occurs with a formation of Li_*x*_Si_*y*_C and Si, followed by a reversible alloying/dealloying reaction of the Si with Li^+^. This irreversible reaction of the SiC anode is responsible for its lower coulombic efficiency than the Si anode in the initial cycles. In addition, due to the low activity of the SiC anode, residual SiC component exists in the SiC anode even after repeated electrochemical cycling. This residual SiC reduces the specific capacity of the anode. On the other hand, this residual SiC can act as a buffer matrix surrounding the active materials to alleviate the volume change, thus improving the mechanical stability and capacity retention of the SiC anode compared with the Si one.

## Conflicts of interest

There are no conflicts to declare.

## Supplementary Material

RA-008-C7RA12463E-s001
