# Supplementary material for: Electrochemical characteristics of amorphous silicon carbide film as a lithium-ion battery anode
Source: RSC Adv. 2018 Jan 30;8(10):5189–96. doi: 10.1039/c7ra12463e (PMC9078100; doi:10.1039/c7ra12463e)
Supplement: RA-008-C7RA12463E-s001 [file RA-008-C7RA12463E-s001.pdf]

Electronic Supplementary Material (ESI) for RSC Advances.
This journal is © The Royal Society of Chemistry 2018

Supplementary Information

Electrochemical characteristics of silicon carbide film as lithium-ion battery anode

X.D. Huang [a*], F. Zhang[a], X.F. Gan[a], Q.A. Huang[a], J.Z. Yang[b*], P.T. Lai[c] and W.M. Tang[d*]

[a]*Key Laboratory of MEMS of the Ministry of Education, Southeast University, Nanjing 210096, China*

[b]*School of Chemistry and Chemical Engineering, Nanjing University of Science and Technology, Nanjing 210094, China*

[c]*Department of Electrical and Electronic Engineering, the University of Hong Kong, Hong Kong, China*

[d]*Department of Applied Physics, the Hong Kong Polytechnic University, Hong Kong, China*

[*] Electronic mail: xdhuang@seu.edu.cn, jiazhiyang@sina.com, wm.tang@polyu.edu.hk

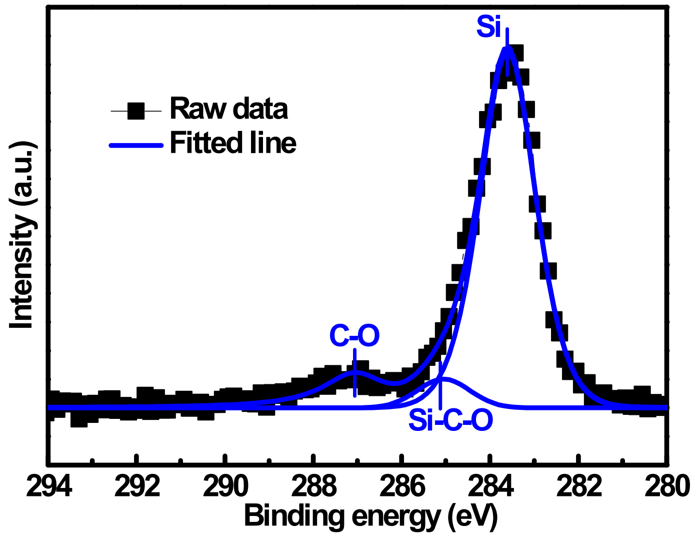

Fig. S1. C 1s XPS spectrum of the 500-nm fresh SiC sample. Note that the sample is etched by Ar^{+} sputtering for 1 min before test.

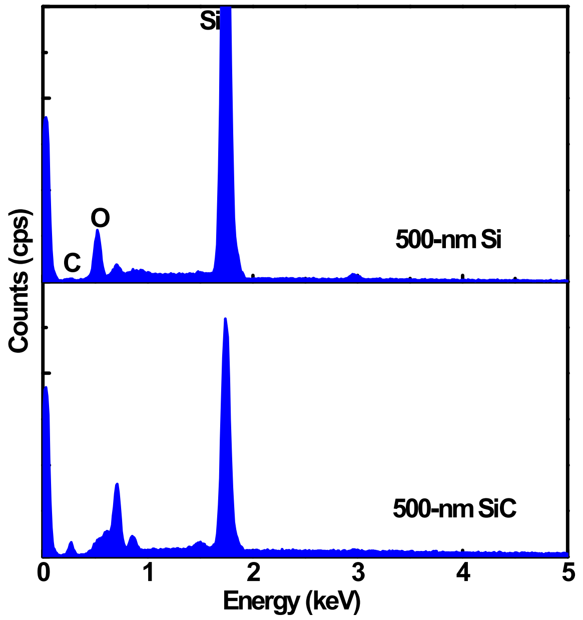

Fig. S2. EDX of the fresh Si and SiC samples, where the peaks from the substrate (including Fe, Cr, Mn and Ni) are not labeled.

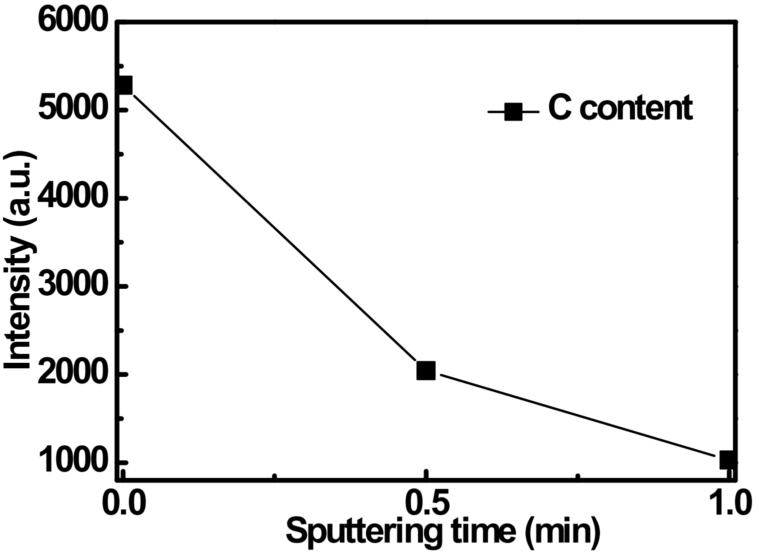

Fig. S3. C 1s XPS depth profiling of the fresh Si sample as a function of Ar^+ sputtering time.
